# X‐linked ichthyosis: New insights into a multi‐system disorder

**DOI:** 10.1002/ski2.179

**Published:** 2022-10-17

**Authors:** Georgina H. Wren, William Davies

**Affiliations:** ^1^ School of Psychology Cardiff University Cardiff UK; ^2^ School of Medicine Cardiff University Cardiff UK; ^3^ Centre for Neuropsychiatric Genetics and Genomics Cardiff University Cardiff UK; ^4^ Neuroscience and Mental Health Innovation Institute Cardiff University Cardiff UK

## Abstract

**Background:**

X‐linked ichthyosis (XLI) is a rare genetic condition almostexclusively affecting males; it is characterised by abnormal desquamation and retentionhyperkeratosis, and presents with polygonal brown scales. Most cases resultfrom genetic deletions within Xp22.31 spanning the STS (steroid sulfatase)gene, with the remaining cases resulting from STS‐specific mutations. For manyyears it has been recognised that individuals with XLI are at increased risk ofcryptorchidism and corneal opacities.

**Methods:**

We discuss emerging evidence that such individuals are alsomore likely to be affected by a range of neurodevelopmental and psychiatrictraits, by cardiac arrhythmias, and by rare fibrotic and bleeding‐relatedconditions. We consider candidate mechanisms that may confer elevatedlikelihood of these individual conditions, and propose a novel commonbiological risk pathway.

**Results:**

Understanding the prevalence, nature and co‐occurrence ofcomorbidities associated with XLI is critical for ensuring early identificationof symptoms and for providing the most effective genetic counselling andmultidisciplinary care for affected individuals.

**Conclusion:**

Future work in males with XLI, and in new preclinical andcellular model systems, should further clarify underlying pathophysiologicalmechanisms amenable to therapeutic intervention.

## Graphical abstract

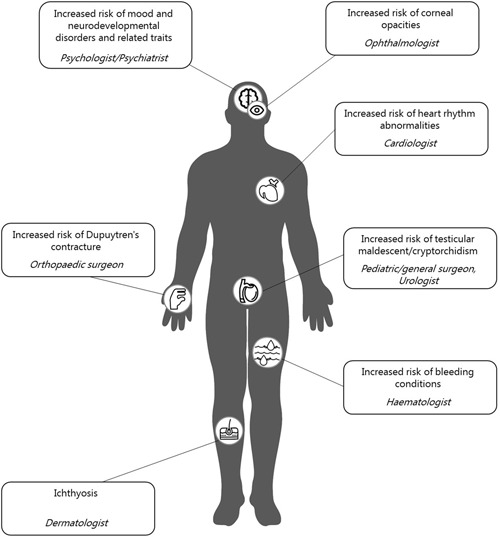

X‐linked ichthyosis is a rare genetic skin condition. We discuss here how it is also associated with multiple medical conditions which may impact affected individuals&apos; education, quality of life and mortality. Awareness and understanding of these comorbidities will be important for ensuring the best multidisplinary care for individuals with XLI.

1

**What's already known about this topic?**

- X‐linked ichthyosis (XLI) has long been associated with an increased risk of testicular maldescent and corneal opacities.
- Historic use of comparatively small samples of young participants has limited our ability to detect comorbidities of medical significance.

**What does this study add?**

- Recent larger‐scale studies including older participants have uncovered new clinically‐significant medical conditions associated with XLI, and point to common pathophysiological mechanisms.
- Understanding the prevalence and nature of conditions associated with XLI will ensure optimal genetic counselling and multidisciplinary care for affected individuals, and will signpost new research avenues in patients and model systems.

## 2 X‐LINKED ICHTHYOSIS

The ichthyoses are dermatological conditions arising from abnormal cornification and desquamation processes, and characterised by dry, thickened scales.^1^ In the early 20th Century, an ichthyosis subtype inherited as an X‐linked recessive trait (i.e., transmitted from unaffected female carriers to sons) was identified.^2^ Biochemical studies in skin fibroblasts of affected individuals correctly predicted deficiency for the enzyme steroid sulfatase (STS) as a causal mechanism,^3^ and the *STS* gene was subsequently cloned.^4^ STS cleaves sulfate groups from multiple steroid hormones (e.g., dehydroepiandrosterone sulfate, DHEAS), affecting their water‐solubility, bioavailability, and activity.^5^ The central mechanism behind the skin phenotype in ‘X‐linked recessive ichthyosis’ (XLI) is probably an accumulation of cholesterol sulfate (and a deficit of cholesterol) in the stratum corneum.^6^, ^7^

Clinically, XLI presents from birth (or shortly afterwards) with widely‐distributed polygonal, translucent scales which are gradually replaced with large, darker brown‐grey scales occurring primarily on the neck, trunk, and lower extremities, and on extensor surfaces.^8^ Most affected males inherit an Xp22.31 genetic deletion from a heterozygous carrier mother; this can be *STS*‐specific, but is typically 1.5–1.7Mb in size, encompassing *STS* and its immediate neighbours (the protein‐coding genes *PUDP*(*HDHD1*), *VCX*, and *PNPLA4* and the non‐coding microRNA *MIR4767*).^8^, ^9^, ^10^, ^11^ In the remaining XLI cases, the causal variant is an *STS* point mutation or, rarely, a larger deletion covering many contiguous genes.^8^, ^9^, ^10^, ^11^ Individuals with extensive deletions frequently present with multiple developmental issues (‘syndromal ichthyosis’)^10^; here, we largely focus on typical ‘non‐syndromic’ XLI cases.

Prenatal screening studies estimate typical Xp22.31 deletions to be present in 1 in 1500 general population males,^12^, ^13^ yet XLI is diagnosed in as few as 1 in 6000 males.^8^ This implies STS deficiency is associated with a spectrum of skin disease, with many carriers either not receiving an XLI diagnosis, or being misdiagnosed. Consistent with this, individuals ascertained genetically present with less severe skin phenotypes than those ascertained in dermatology clinics.^14^ Moreover, <60% of phenotypically‐characterised males with Xp22.31 deletions <10Mb around *STS* reported in the DECIPHER clinical genetics database (the ‘DECIPHER XLI‐relevant cohort’) are identified as having ichthyosis or a ‘skin abnormality’.^15^ The severity of the skin condition in XLI may be modified by an individual's background genetics, notably variants within the autosomal *FLG* (filaggrin) gene.^16^

As Xp22.31 gene products are widely‐expressed in the human body,^17^ their deficiency may be associated with extracutaneous medical phenotypes. Recognition of such phenotypes has been hampered by the rarity of XLI, superfluous phenotyping of cases, and ascertainment biases (cases have largely been identified in dermatology clinics on the basis of their moderate‐severe skin condition, and are often young). Over the past decade, improved recruitment across the age range, genotyping/phenotyping and data collation and dissemination strategies (particularly through large‐scale biobanks and clinical genetics resources), has revealed new genotype‐phenotype associations.

## 3 ESTABLISHED MEDICAL COMORBIDITIES

### 3.1 Cryptorchidism

Case reports/series published in the late 1970s and early 1980s highlighted a putative association between XLI and bilateral or unilateral cryptorchidism (testicular maldescent into the scrotum during development)^18^; these early studies indicated cryptorchidism in 10%–40% of individuals with XLI, notably in those whose birth was beset by obstetric complications (placental STS deficiency delays or prolongs labour in >60% of carrier mothers^19^). More recent case series,^20^, ^21^ and the DECIPHER XLI‐relevant cohort,^15^ suggest a cryptorchidism prevalence rate in XLI of around 10%–15%, consistent with the lower end of this initially‐predicted range, and higher than the 2%–8% prevalence in the general pediatric population.^22^ Biological explanations for increased cryptorchidism risk include: deficiency for Xp22.31 gene product(s), prepubertal hormonal perturbations downstream of STS deficiency (e.g., elevated steroid sulfate or luteinizing hormone levels), or disruption of local chromosomal architecture by the genetic deletion and mis‐expression of contiguous genes.^18^, ^23^ Despite this apparent vulnerability to structural gonadal abnormalities, the majority of individuals with XLI have preserved fertility and normal sexual development^8^; serum testosterone levels in boys with XLI are equivalent to those in non‐affected boys developmentally, but tend to be lower post‐pubertally.^23^

### 3.2 Corneal opacities

In the early‐mid 1980s, it was recognized that many males with XLI (and female carriers) presented with corneal dystrophy observable as a ‘frosted layer’, or small punctate/filiform inclusions usually located deep in the posterior corneal stroma adjacent to, or within, the Descemet basement membrane.^8^, ^24^, ^25^, ^26^ These proteinaceous bodies may arise as consequence of locally‐elevated cholesterol sulfate levels.^27^ The opacities tend to manifest in adolescence or early adulthood and do not seem to impede visual acuity, although they have occasionally been linked to corneal erosion.^8^ Estimates suggest that opacities may be more common in males with XLI (prevalence 10%–15%)^8^ than in the general population of USA (<7.5%).^28^

### 3.3 Conditions co‐occurring with XLI in rare cases

Historically, a range of syndromes and medical conditions (notably testicular germ cell cancer^29^) have been described in very few XLI cases.^21^ The rarity of XLI, and often of the comorbid disorders too, makes identification of any consistent relationships challenging.

## 4 NEWLY‐IDENTIFIED MEDICAL COMORBIDITIES

### 4.1 Neurodevelopmental conditions

#### 4.1.1 Learning disability

After ichthyosis, cognitive impairments (intellectual disability, global developmental delay and delayed speech/language development) are the most commonly‐described phenotype in the DECIPHER XLI‐relevant cohort, even amongst carriers of typically‐sized deletions.^15^ However, XLI has not generally been associated with large effects on cognition, and we have shown using the large UK Biobank (UKBB) resource that, whilst middle‐aged males carrying typical XLI‐associated genetic deletions performed marginally worse than non‐carrier males on a fluid intelligence task, the former group performed equivalently to the latter on most other cognitive tasks, and with respect to academic achievement.^9^ However, it should be noted that UKBB is depleted for individuals with neurodevelopmental and/or psychiatric conditions,^30^ and ∼30% of eligible individuals carrying typical XLI‐associated deletions may not have been recruited into UKBB (perhaps, in part due to psychological issues).^9^ Hence, the cognitive effects of XLI‐associated deletions may be somewhat larger than our UKBB analysis suggests. There have been case reports in the literature of individuals with XLI and learning disabilities; in these cases, the genetic deletions tend to be larger, and often encompass the *VCX3A* (formerly *VCXA*) and/or *NLGN4X* genes.^20^, ^31^, ^32^, ^33^, ^34^ Lack of VCX3A and/or NLGN4X proteins may confer vulnerability to neurodevelopmental conditions,^35^, ^36^, ^37^, ^38^ but their deficiency does not inevitably result in gross effects on cognition.^39^, ^40^ Overall, these data suggest that typical XLI‐associated deletions alone predispose to mild general cognitive impairment at most, and that the moderate‐severe learning disabilities seen infrequently in individuals with XLI (such as those referred to genetics clinics and potentially excluded from UKBB) may be explained by a combination of additional factors: variably‐penetrant deletion of adjacent Xp22.31 genes, the extent/nature of local chromatin disruption, co‐segregating genetic variants, environmental exposures and stochastic developmental processes.

The interpretation above is supported by the finding that STS‐deficient animal models exhibit normal learning of complex cognitive tasks.^41^, ^42^, ^43^ Intriguingly, work in such models has shown that deletion of the *STS* orthologue, or inhibition of the STS enzyme, can actually enhance aspects of memory, alter hippocampal neurochemistry, protect against neurodegenerative disease‐associated pathology, and increase longevity.^44^, ^45^, ^46^, ^47^, ^48^ Hippocampal volume is comparable in Xp22.31 deletion carriers and non‐carriers,^9^ but whether males with XLI exhibit altered hippocampal function, and protection against age‐related pathology, is worth investigating in future work.

#### 4.1.2 Attention deficit hyperactivity disorder

Although attention impairments and hyperactivity were reported in rare *STS*‐deficient cases with chromosomal rearrangements in early 2000s,^49^, ^50^ it was not until 2008 that the first case series examining Attention deficit hyperactivity disorder (ADHD) in XLI was described.^33^ 40% of boys with XLI assessed met diagnostic criteria for the condition, 80% with the inattentive subtype; the prevalence of ADHD in males from the general population is far lower (≤5%).^51^ Later case series across different countries have confirmed that around 30% of boys with XLI meet diagnostic criteria for ADHD, with several co‐presenting with other neurodevelopmental conditions including Tourette syndrome, dyspraxia and epilepsy.^20^, ^21^ An online survey comparing self‐reported ADHD diagnoses/related traits in males with XLI to those in matched controls confirmed an excess of most traits (apart from ‘motor impulsivity’) in the former group,^52^ a pattern of results recapitulated in female carriers.^19^ It is important for individuals affected by XLI and medical professionals to be aware that high levels of neurodevelopmental (or psychiatric) traits in the absence of significant functional impairment can be advantageous and contribute to population neurodiversity.^53^

Neuroimaging data on individuals with XLI are sparse, and the neurobiological mechanisms mediating increased ADHD likelihood require investigation. Cases with larger (8.4Mb)^32^ and typically‐sized deletions^54^, ^55^ exhibit cortical malformations (heterotopia/dysplasia), but cortical morphology has yet to be systematically investigated. Volumetric analysis of subcortical regions suggests that the smaller size of basal ganglia sub‐regions important in maintaining focus and regulating impulse control in deletion carriers may be relevant.^9^ Numerous lines of evidence implicate *STS* specifically in ADHD‐related endophenotypes: (a) individuals with gene‐specific point mutations meet diagnostic criteria,^33^ (b) the gene is highly‐expressed in the developing basal ganglia,^56^ (c) within‐gene variation is associated with (in)attentive (but not impulsivity) symptoms in boys with ADHD^56^ and healthy men,^57^ (d) independent mouse models lacking STS indicate impairments in attention,^43^, ^58^ reduced motor impulsivity,^41^ and altered basal ganglia serotonergic neurochemistry,^59^ and (e) circulating DHEA levels inversely correlate with symptom severity^60^ and are sensitive to pharmacotherapy.^61^ The neurobehavioural features and executive function deficits seen in some individuals with XLI could be exacerbated by poor sleep quality (due to night‐time discomfort/itching, stress, or aberrant temperature regulation^62^, ^63^) and, if so, might be ameliorated through sleep‐based interventions.

#### 4.1.3 Autism

Phenotype‐first approaches have highlighted a preponderance of *STS*‐spanning deletions in people with autism.^64^ In Kent and colleagues' genotype‐first approach,^33^ boys with XLI were also screened for autism. 20% of participants met diagnostic criteria for this condition or a related language/communication difficulty, a figure markedly higher than the <5% male general population prevalence.^65^ Affected boys possessed larger genetic deletions including the *VCX3A* and *NLGN4X* genes and so deficiency for one (or both) of these genes was suggested as being causal for their behavioural phenotype; however, again, it is possible that other genetic/environmental factors co‐segregating with the Xp22.31 deletion are responsible. XLI cases with the typical deletion can present with autism,^66^ and self‐reported autism‐related traits in males with XLI^52^ and female carriers^19^ generally appear to be higher than in matched general population control subjects, implying that loss of gene(s) within the typical deletion interval predisposes to autism‐related traits. In both males with XLI^52^ and female carriers^19^ the only autism‐related trait which is not elevated compared to matched controls is ‘attention to detail’. Differing basal ganglia morphology/neurochemistry could partially explain the higher frequency of autism‐related traits in individuals with XLI.^9^ STS‐deficient mice exhibit autism‐like features (e.g., perseverative behaviour) highlighting *STS* as a credible gene influencing autism‐related traits.^42^

#### 4.1.4 Epilepsy

10–15% of individuals with typical XLI deletions have been reported to develop a treatable childhood‐onset form of epilepsy; this often presents as focal epilepsy with centrotemporal spikes.^21^, ^54^, ^67^ The prevalence of epilepsy in boys from the general population is <1%.^68^ In deletion carriers, epilepsy is frequently comorbid with other neurodevelopmental conditions (notably ADHD) suggesting a common cause. Individuals with comorbid XLI and epilepsy have presented with cortical dysplasia^54^ or periventricular leukomalacia,^67^ although others display no clear neuroanatomical abnormalities.^54^ STS deficiency appears the prime candidate mechanism for epilepsy predisposition given the STS axis' role in modulation of relevant neurotransmitter receptors,^69^ and that a potentially‐pathogenic *STS* point mutation has been observed in two brothers with epilepsy.^70^ However, epilepsy‐related symptoms have not been observed in STS‐deficient rodents, so deficiency for alternative Xp22.31 products^54^ or co‐segregating factors may confer risk.

#### 4.1.5 Schizophrenia/psychosis

Genetic risk variants can act pleiotropically to influence both early‐onset neurodevelopmental conditions and later‐onset psychiatric conditions including schizophrenia.^71^, ^72^ Xp22.31 deletion may elevate schizophrenia risk, particularly against a background of other neurodevelopmental conditions: a young boy with a typical deletion presented with psychotic symptoms consistent with early‐onset schizophrenia,^67^ and two females presented with paranoid schizophrenia.^73^ Additionally, female carriers endorse more schizotypal personality traits than matched non‐carriers,^19^ and DHEAS levels positively correlate with lifetime psychotic symptom probability in a patient cohort.^74^ Again, STS deficiency appears a plausible mechanism.^75^

### 4.2 Mood disorders

A convincing association between XLI and elevated mood disorder diagnoses/traits (depression, anxiety and irritability) of comparable magnitude to that seen for psoriasis has recently been demonstrated.^9^, ^52^, ^76^, ^77^ Lower basal ganglia volume, disrupted serotonergic function, and/or altered steroid hormone levels represent candidate biological risk mechanisms.^9^ Putative ‘environmental risk’ mechanisms include: patient concerns about their appearance and bullying/stigmatisation, the need to manage treatments, side‐effects of retinoid‐derived medications, medical complications associated with the condition, sleep disturbance, or educational/social challenges linked to neurodevelopmental issues.^77^ Men with XLI perceive the first two of these as being the most strongly‐linked to adverse mood symptoms; interventions to improve societal knowledge of ichthyosis and thereby destigmatise it, together with advice related to treatment sourcing/application, and a holistic approach to multidisciplinary care, would likely mitigate mood issues.^77^

### 4.3 Cardiac arrhythmias

Middle‐aged males carrying typical XLI‐associated deletions are around four times more likely to have been diagnosed with atrial fibrillation/flutter (AF) than their non‐carrier counterparts (10.5% vs. 2.7% of UKBB participants respectively),^9^ and AF affected one middle‐aged male with XLI described in a recent case report.^78^ AF is a type of irregular heart rhythm which increases heart failure, stroke, and dementia risk, and, as such requires early identification, monitoring and treatment.^79^ The higher burden of cardiac arrhythmias in deletion carriers could, theoretically, be due to side‐effects of medication use specific to this group for example, retinoid‐based medications for ichthyosis or stimulant medications for ADHD.^80^ Use of these medications in UKBB sample was very low and did not differ by group,^9^ and there is little evidence that ichthyosis treatments promote arrhythmia, so a biological predisposition to cardiovascular risk appears more likely. Structural heart abnormalities, but not arrhythmias, have been reported in 7% of the predominantly‐young DECIPHER XLI‐relevant cohort (ventricular‐septal defect and abnormal valve morphology),^15^ and an abnormal heart rhythm has only been reported in one boy with XLI.^81^ Cardiac arrhythmias might manifest in adulthood in deletion carriers with underlying structural heart issues.

Our ongoing (unpublished) work in UKBB indicates that males with XLI and abnormal heart rhythms are disproportionately likely to be affected by gastrointestinal issues, and that *STS* is the only protein‐coding Xp22.31 gene harbouring an excess of common risk variants for idiopathic AF. The finding that cancer patients pre‐screened for existing heart rhythm anomalies occasionally present with these following STS inhibition^82^ further supports STS deficiency as a mechanism for arrhythmia risk. STS deficiency could feasibly mediate risk via effects on cardiovascular circulation, DHEA(S) levels, cholinergic signalling and/or fibrosis.^79^

### 4.4 Dupuytren's contracture

Dupuytren's contracture, in which one or more fingers become permanently flexed, typically onsets in middle‐age due to fibrosis of the palmar fascia.^83^ In UKBB, we identified diagnosis in 3.5% of male deletion carriers (compared to 0.6% in non‐carriers),^84^ and the DECIPHER database describes one patient with a pathogenic point mutation within *STS* and contracture of the 5th finger.^15^

### 4.5 Bleeding conditions

Work in UKBB also identified a specific bleeding‐related phenotype (‘hemorrhage or hematoma complicating a procedure’) as being more common (3.5%) in deletion‐carrying males than male non‐carriers (0.5%).^84^ This finding could reflect the fact that deletion carriers undergo more, or more invasive, procedures than non‐carriers and/or that deletion carriers are more likely to be prescribed retinoid‐derived pharmacotherapy with adverse effects on blood‐clotting.^85^ Alternatively, and perhaps more likely given that no deletion carriers reported being prescribed such medications, it could have a biological explanation. Relevantly, *STS* is most highly‐expressed in the adult arterial vasculature^17^ and cholesterol sulfate is a known endogenous haemostatic modulator.^86^

## 5 A LINK BETWEEN COMORBIDITIES?

The conditions linked to XLI above appear disparate in their presentation, and in the bodily systems they affect. Is it the case that a common pathophysiological process, induced by deficiency for one or more Xp22.31 gene(s), is responsible, and, if so, could this be targeted pharmacologically?

Few data currently exist on the molecular pathways disrupted in vivo by XLI. One study comparing skin gene expression in affected and unaffected individuals identified just 6 genes outside the Xp22.31 region the expression of which differed by > 3‐fold: *GDA*, *UPK1A*, *LAMC1*, *PPME1*, *KLK9* and *MLL5*.^87^ *LAMC1* encodes the gamma 1 subunit of the heterotrimeric laminin complex, a critical constituent of the extracellular matrix (ECM) basement membrane to which the interstitial ECM adheres.^88^

On the basis of multiple lines of evidence (Table 1), we propose that extracutaneous phenotypes associated with XLI may be partially explained by perturbed ECM function, and specifically fundamental changes to laminin expression and basement membrane integrity. Subsequent disruption to cross‐talk between basement membrane and matricellular proteins (such as the Cellular Communication Network factors (CCNs) known to be sensitive to STS deficiency^109^) might predispose to (extra)cellular pathophysiology including fibrosis.

**TABLE 1:** Lines of evidence supporting the role of altered laminin and basement membrane function in X‐linked ichthyosis (XLI) extracutaneous phenotypes

Extracutaneous phenotype	Evidence implicating laminin and/or basement membrane pathology
Cryptorchidism	*Lamc1* (and *Ccn1*/*Cyr61*) identified as hub genes by gene expression analysis in a rat model^89^
Testicular germ cell cancer	Zebrafish mutant for *Lamc1* susceptible to spontaneous and carcinogen‐induced testicular germ cell tumours^90^
Corneal opacity	Descemet and epithelial basement membranes in the cornea affected in XLI.^8^, ^25^, ^91^ *LAMC1* genetically‐associated with Fuch's endothelial corneal dystrophy, a condition linked to thickening of the Descemet membrane due to excessive extracellular matrix deposition^92^
Neurodevelopmental conditions	LAMC1 mediates cortical histogenesis and its abnormal expression results in disrupted basement membrane structure and cortical dysplasia.^93^ Laminin protein disruption associated with cortical heterotopias in man^94^. *LAMC1* shows genetic association with cortical folding and thickness,^95^, ^96^ the latter robustly differentiating ADHD, autism and schizophrenia cases from controls^97^, ^98^
Cardiac arrhythmia	General basement membrane dysfunction implicated in cardiovascular pathologies including arrhythmia.^99^ Laminin (including LAMC1) protein levels differ in patients with and without atrial fibrillation.^100^, ^101^ *LAMC1* gene implicated in arrhythmia.^102^ Laminins (including LAMC1) important in regulation of heart looping, atrial growth and cardiac size during development.^103^ *LAMC1*‐mutant cardiomyocyte cultures display impaired electrical signal propagation^104^
Dupuytren's contracture	Disrupted basal lamina structure, and high laminin (including LAMC1) and CTGF(CCN2) expression, in palmar fascial nodules^105^, ^106^, ^107^
Hemorrhage	Hemorrhage risk increased in *Lamc1* mutant mice due to impaired function of the basement membrane ensheathing the vasculature^93^, ^108^

## 6 CONCLUSIONS

**FIGURE 1:**
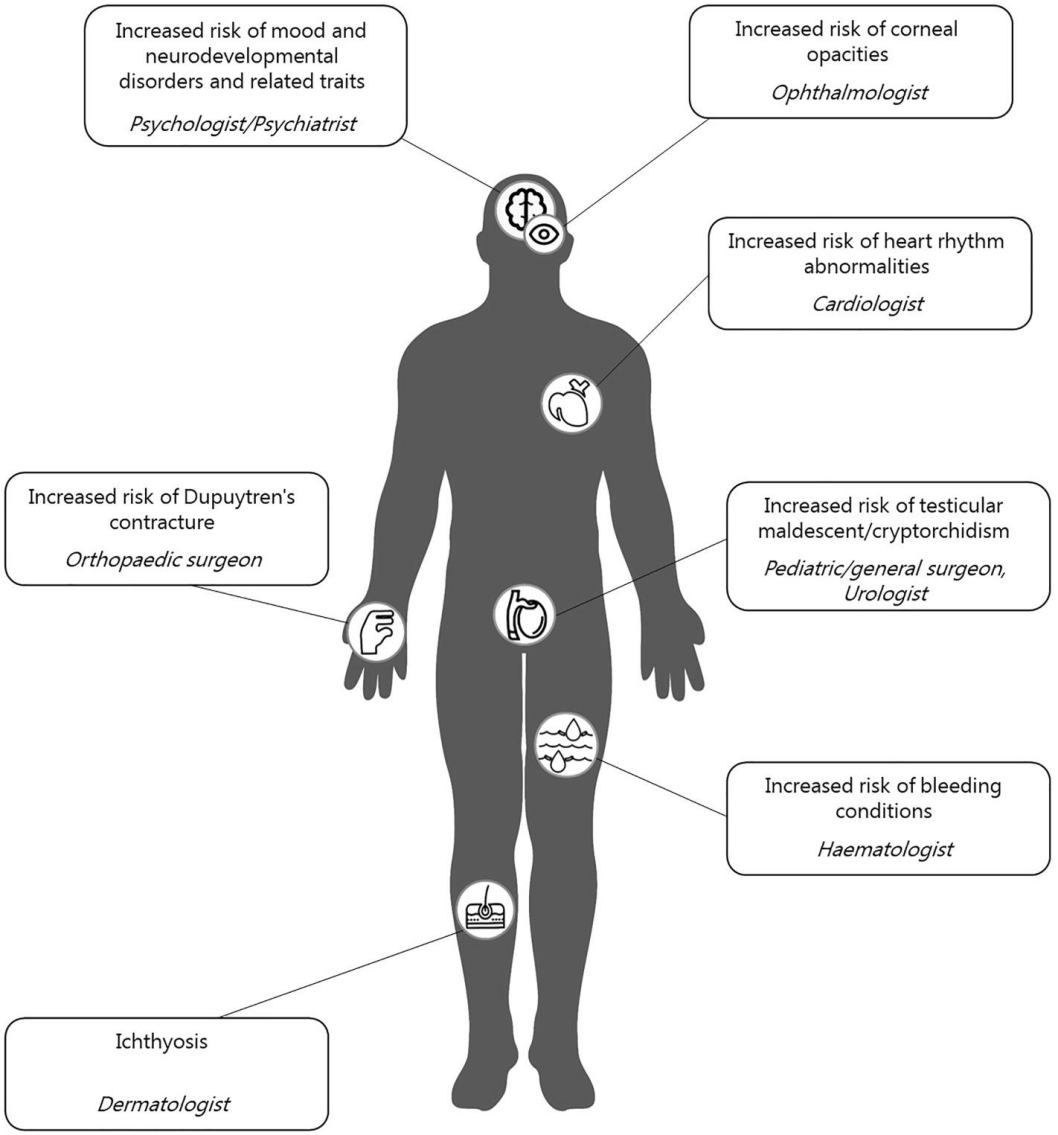
A summary of cutaneous and extracutaneous symptoms associated with X‐linked ichthyosis (XLI), and clinicians relevant for multidisciplinary care of affected individuals.

Individuals with XLI are at greater likelihood of being affected by various medical conditions, which can impact their education, quality of life, morbidity and mortality (Figure 1). Future high‐powered studies in geographically/ethnically‐diverse populations should aim to replicate, extend, and further characterise, the associations discussed above. Specifying the prevalence, nature and co‐occurrence of comorbidities and disseminating findings to individuals affected by XLI and their family members, treating clinicians, and genetic counsellors should facilitate early detection of problems and referral to appropriate medical specialists (Figure 1), with ensuing benefits in terms of treatment efficacy.

**FIGURE 2:**
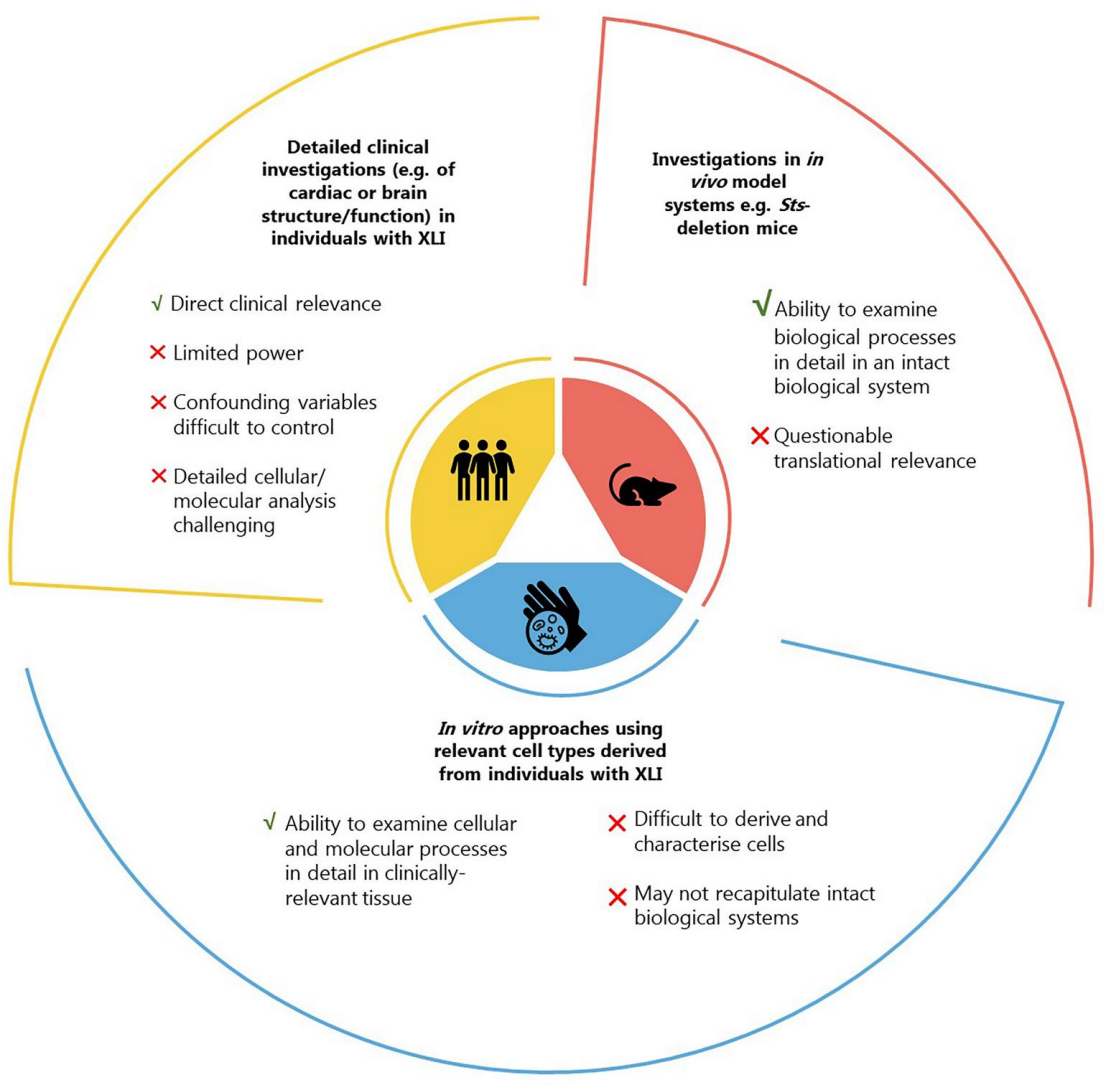
Potential approaches to understand the pathophysiology of extracutaneous phenotypes associated with X‐linked ichthyosis (XLI).

Understanding the physiological, cellular and molecular processes underlying XLI comorbidities will require work across experimental paradigms (Figure 2). Detailed phenotyping in individuals with XLI might include psychiatric/neuropsychological evaluations, imaging and monitoring of electrical signals of the brain and heart, and multi‐omic analysis of accessible tissues; however, recruiting sufficient participants to ensure adequate power and to address confounds may be challenging. In vitro cellular or organoid models with engineered *STS* mutations, exposed to *STS*‐knockdown, or derived from patient tissues, might be useful to examine condition‐related cell biology processes, but their generation can be challenging and findings may not reflect whole organism physiology. Arguably, investigations in preclinical models in which the *Sts* gene is specifically targeted may be the most appropriate way to address multisystemic conditions, although given species differences in steroid hormone biochemistry, and organ structure/function, their translational relevance is questionable. Should (pre)clinical or cellular studies uncover support for abnormal ECM function in XLI, laminin and CCN proteins could represent viable pharmacotherapeutic targets.^110^, ^111^, ^112^

## CONFLICT OF INTEREST

The authors declare that there is no conflict of interest that could be perceived as prejudicing the impartiality of the research reported.

## AUTHOR CONTRIBUTIONS

**Georgina H. Wren**: Visualization (lead); Writing – original draft (supporting); Writing – review & editing (equal). **William Davies**: Conceptualization (lead); Funding acquisition (lead); Supervision (lead); Writing – original draft (lead); Writing – review & editing (equal).

## ETHICS STATEMENT

Not applicable.

## Data Availability

Data sharing not applicable—no new data generated.

## References

[1] Park JS , Saeidian AH , Youssefian L , Kondratuk KE , Pride HB , Vahidnezhad H , et al. Inherited ichthyosis as a paradigm of rare skin disorders: genomic medicine, pathogenesis, and management. J Am Acad Dermatol. 2022. 10.1016/j.jaad.2022.08.012 35963288

[2] Cockayne EA . Inherited abnormalities of the skin and its appendages. Oxford University Press; 1933.

[3] Webster D , France JT , Shapiro LJ , Weiss R . X‐linked ichthyosis due to steroid‐sulphatase deficiency. Lancet. 1978;1(8055):70–2. 10.1016/s0140-6736(78)90005-3 74568

[4] Yen PH , Marsh B , Allen E , Tsai SP , Ellison J , Connolly L , et al. The human X‐linked steroid sulfatase gene and a Y‐encoded pseudogene: evidence for an inversion of the Y chromosome during primate evolution. Cell. 1988;55(6):1123–35. 10.1016/0092-8674(88)90257-7 3203382

[5] Mueller JW , Gilligan LC , Idkowiak J , Arlt W , Foster PA . The regulation of steroid action by sulfation and desulfation. Endocr Rev. 2015;36(5):526–63. 10.1210/er.2015-1036 26213785 PMC4591525

[6] Fandrei F , Engberg O , Opalka L , Jancalkova P , Pullmannova P , Steinhart M , et al. Cholesterol sulfate fluidizes the sterol fraction of the stratum corneum lipid phase and increases its permeability. J Lipid Res. 2022;63(3):100177. 10.1016/j.jlr.2022.100177 35143845 PMC8953687

[7] Elias PM , Williams ML , Choi EH , Feingold KR . Role of cholesterol sulfate in epidermal structure and function: lessons from X‐linked ichthyosis. Biochim Biophys Acta. 2014;1841(3):353–61. 10.1016/j.bbalip.2013.11.009 24291327 PMC3966299

[8] Fernandes NF , Janniger CK , Schwartz RA . X‐linked ichthyosis: an oculocutaneous genodermatosis. J Am Acad Dermatol. 2010;62(3):480–5. 10.1016/j.jaad.2009.04.028 20080321

[9] Brcic L , Underwood JF , Kendall KM , Caseras X , Kirov G , Davies W . Medical and neurobehavioural phenotypes in carriers of X‐linked ichthyosis‐associated genetic deletions in the UK Biobank. J Med Genet. 2020;57(10):692–98. 10.1136/jmedgenet-2019-106676 32139392 PMC7525778

[10] Fischer J , Bourrat E . Genetics of inherited ichthyoses and related diseases. Acta Derm Venereol. 2020;100(7):adv00096–196. 10.2340/00015555-3432 32147747 PMC9128940

[11] Cuevas‐Covarrubias SA , Valdes‐Flores M , Orozco Orozco E , Diaz‐Zagoya JC , Kofman‐Alfaro SH . Most ‘sporadic’ cases of X‐linked ichthyosis are not de novo mutations. Acta Derm Venereol. 1999;79(2):143–4. 10.1080/000155599750011381 10228635

[12] Craig WY , Roberson M , Palomaki GE , Shackleton CHL , Marcos J , Haddow JE . Prevalence of steroid sulfatase deficiency in California according to race and ethnicity. Prenat Diagn. 2010;30(9):893–8. 10.1002/pd.2588 20715120

[13] Langlois S , Armstrong L , Gall K , Hulait G , Livingston J , Nelson T , et al. Steroid sulfatase deficiency and contiguous gene deletion syndrome amongst pregnant patients with low serum unconjugated estriols. Prenat Diagn. 2009;29(10):966–74. 10.1002/pd.2326 19609942

[14] Hand JL , Runke CK , Hodge JC . The phenotype spectrum of X‐linked ichthyosis identified by chromosomal microarray. J Am Acad Dermatol. 2015;72(4):617–27. 10.1016/j.jaad.2014.12.020 25659225

[15] DECIPHER v11.14 Mapping the clinical genome . Available from: https://www.deciphergenomics.org/. Accessed 7 September 2022.

[16] Sussmuth K , Gruber R , Rodriguez E , Traupe H , Amler S , Sanchez‐Guijo A , et al. Increased prevalence of filaggrin deficiency in 51 patients with recessive X‐linked ichthyosis presenting for dermatological examination. J Invest Dermatol. 2018;138(3):709–11. 10.1016/j.jid.2017.08.047 29054605

[17] GTEx Portal . Available from: https://gtexportal.org/home/. Accessed 7 September 2022.

[18] Traupe H , Happle R . Mechanisms in the association of cryptorchidism and X‐linked recessive ichthyosis. Dermatol. 1986;172(6):327–8. 10.1159/000249372 2874062

[19] Cavenagh A , Chatterjee S , Davies W . Behavioural and psychiatric phenotypes in female carriers of genetic mutations associated with X‐linked ichthyosis. PLoS One. 2019;14(2):e0212330. 10.1371/journal.pone.0212330 30768640 PMC6377116

[20] Diociaiuti A , Angioni A , Pisaneschi E , Alesi V , Zambruno G , Novelli A , et al. X‐linked ichthyosis: clinical and molecular findings in 35 Italian patients. Exp Dermatol. 2019;28(10):1156–63. 10.1111/exd.13667 29672931

[21] Rodrigo‐Nicolas B , Bueno‐Martinez E , Martin‐Santiago A , Canueto J , Vicente A , Torrelo A , et al. Evidence of the high prevalence of neurological disorders in nonsyndromic X‐linked recessive ichthyosis: a retrospective case series. Br J Dermatol. 2018;179(4):933–39. 10.1111/bjd.16826 29901853

[22] Virtanen HE , Bjerknes R , Cortes D , Jorgensen N , Rajpert‐De Meyts E , Thorsson AV , et al. Cryptorchidism: classification, prevalence and long‐term consequences. Acta Paediatr. 2007;96(5):611–6. 10.1111/j.1651-2227.2007.00241.x 17462053

[23] Idkowiak J , Taylor AE , Subtil S , O'Neil DM , Vijzelaar R , Dias RP , et al. Steroid sulfatase deficiency and androgen activation before and after puberty. J Clin Endocrinol Metab. 2016;101(6):2545–53. 10.1210/jc.2015-4101 27003302 PMC4891801

[24] Haritoglou C , Ugele B , Kenyon KR , Kampik A . Corneal manifestations of X‐linked ichthyosis in two brothers. Cornea. 2000;19(6):861–3. 10.1097/00003226-200011000-00023 11095067

[25] Macsai MS , Doshi H . Clinical pathologic correlation of superficial corneal opacities in X‐linked ichthyosis. Am J Ophthalmol. 1994;118(4):477–84. 10.1016/s0002-9394(14)75799-x 7943126

[26] Grala PE . Corneal changes in X‐linked ichthyosis. J Am Optom Assoc. 1985;56(4):315–7. 3872899

[27] Kempster RC , Hirst LW , de la Cruz Z , Green WR . Clinicopathologic study of the cornea in X‐linked ichthyosis. Arch Ophthalmol. 1997;115(3):409–15. 10.1001/archopht.1997.01100150411017 9076217

[28] Yuksel E , Hall NE , Singh RB , et al. Corneal opacity in the United States: an epidemiological study from the IRIS Registry. Invest Ophthalmol Vis Sci. 2020;61:3995.

[29] Lykkesfeldt G , Bennett P , Lykkesfeldt AE , Micic S , Svenstrup B , Rorth M , et al. Testis cancer. Ichthyosis constitutes a significant risk factor. Cancer. 1991;67(3):730–4. 10.1002/1097-0142(19910201)67:3<730::aid-cncr2820670333>3.0.co;2-t 1898710

[30] Fry A , Littlejohns TJ , Sudlow C , Doherty N , Adamska L , Sprosen T , et al. Comparison of sociodemographic and health‐related characteristics of UK biobank participants with those of the general population. Am J Epidemiol. 2017;186(9):1026–34. 10.1093/aje/kwx246 28641372 PMC5860371

[31] Ben Khelifa H , Soyah N , Ben‐Abdallah‐Bouhjar I , Gritly R , Sanlaville D , Elghezal H , et al. Xp22.3 interstitial deletion: a recognizable chromosomal abnormality encompassing VCX3A and STS genes in a patient with X‐linked ichthyosis and mental retardation. Gene. 2013;527(2):578–83. 10.1016/j.gene.2013.06.018 23791652

[32] van Steensel MA , Vreeburg M , Engelen J , Ghesquiere S , Stegmann A , Herbergs J , et al. Contiguous gene syndrome due to a maternally inherited 8.41 Mb distal deletion of chromosome band Xp22.3 in a boy with short stature, ichthyosis, epilepsy, mental retardation, cerebral cortical heterotopias and Dandy‐Walker malformation. Am J Med Genet. 2008;146A(22):2944–9. 10.1002/ajmg.a.32473 18925676

[33] Kent L , Emerton J , Bhadravathi V , Weisblatt E , Pasco G , Willatt LR , et al. X‐linked ichthyosis (steroid sulfatase deficiency) is associated with increased risk of attention deficit hyperactivity disorder, autism and social communication deficits. J Med Genet. 2008;45(8):519–24. 10.1136/jmg.2008.057729 18413370

[34] Hosomi N , Oiso N , Fukai K , Hanada K , Fujita H , Ishii M . Deletion of distal promoter of VCXA in a patient with X‐linked ichthyosis associated with borderline mental retardation. J Dermatol Sci. 2007;45(1):31–6. 10.1016/j.jdermsci.2006.10.001 17113756

[35] Daoud H , Bonnet‐Brilhault F , Vedrine S , Demattei MV , Vourc'h P , Bayou N , et al. Autism and nonsyndromic mental retardation associated with a de novo mutation in the NLGN4X gene promoter causing an increased expression level. Biol Psychiatr. 2009;66(10):906–10. 10.1016/j.biopsych.2009.05.008 19545860

[36] Jiao X , Chen H , Chen J , Herrup K , Firestein BL , Kiledjian M . Modulation of neuritogenesis by a protein implicated in X‐linked mental retardation. J Neurosci. 2009;29(40):12419–27. 10.1523/JNEUROSCI.5954-08.2009 19812318 PMC2787249

[37] Laumonnier F , Bonnet‐Brilhault F , Gomot M , Blanc R , David A , Moizard MP , et al. X‐linked mental retardation and autism are associated with a mutation in the NLGN4 gene, a member of the neuroligin family. Am J Hum Genet. 2004;74(3):552–7. 10.1086/382137 14963808 PMC1182268

[38] Fukami M , Kirsch S , Schiller S , Richter A , Benes V , Franco B , et al. A member of a gene family on Xp22.3, VCX‐A, is deleted in patients with X‐linked nonspecific mental retardation. Am J Hum Genet. 2000;67(3):563–73. 10.1086/303047 10903929 PMC1287516

[39] Mochel F , Missirian C , Reynaud R , Moncla A . Normal intelligence and social interactions in a male patient despite the deletion of NLGN4X and the VCX genes. Eur J Med Genet. 2008;51(1):68–73. 10.1016/j.ejmg.2007.11.002 18194880

[40] Macarov M , Zeigler M , Newman JP , Strich D , Sury V , Tennenbaum A , et al. Deletions of VCX‐A and NLGN4: a variable phenotype including normal intellect. J Intellect Disabil Res. 2007;51(5):329–33. 10.1111/j.1365-2788.2006.00880.x 17391250

[41] Davies W , Humby T , Trent S , Eddy JB , Ojarikre OA , Wilkinson LS . Genetic and pharmacological modulation of the steroid sulfatase axis improves response control; comparison with drugs used in ADHD. Neuropsychopharmacology. 2014;39(11):2622–32. 10.1038/npp.2014.115 24842408 PMC4140762

[42] Trent S , Dean R , Veit B , Cassano T , Bedse G , Ojarikre OA , et al. Biological mechanisms associated with increased perseveration and hyperactivity in a genetic mouse model of neurodevelopmental disorder. Psychoneuroendocrinology. 2013;38(8):1370–80. 10.1016/j.psyneuen.2012.12.002 23276394 PMC3690523

[43] Davies W , Humby T , Kong W , Otter T , Burgoyne PS , Wilkinson LS . Converging pharmacological and genetic evidence indicates a role for steroid sulfatase in attention. Biol Psychiatr. 2009;66(4):360–7. 10.1016/j.biopsych.2009.01.001 PMC2720459 19251250

[44] Vitku J , Hill M , Kolatorova L , Kubala Havrdova E , Kancheva R . Steroid sulfation in neurodegenerative diseases. Front Mol Biosci. 2022;9:839887. 10.3389/fmolb.2022.839887 35281259 PMC8904904

[45] Perez‐Jimenez MM , Monje‐Moreno JM , Brokate‐Llanos AM , Venegas‐Caleron M , Sanchez‐Garcia A , Sansigre P , et al. Steroid hormones sulfatase inactivation extends lifespan and ameliorates age‐related diseases. Nat Commun. 2021;12(1):49. 10.1038/s41467-020-20269-y 33397961 PMC7782729

[46] Yue XH , Tong JQ , Wang ZJ , Zhang J , Liu X , Liu XJ , et al. Steroid sulfatase inhibitor DU‐14 protects spatial memory and synaptic plasticity from disruption by amyloid beta protein in male rats. Horm Behav. 2016;83:83–92. 10.1016/j.yhbeh.2016.05.019 27222435

[47] Johnson DA , Wu T , Li P , Maher TJ . The effect of steroid sulfatase inhibition on learning and spatial memory. Brain Res. 2000;865(2):286–90. 10.1016/s0006-8993(00)02372-6 10821934

[48] Rhodes ME , Li PK , Burke AM , Johnson DA . Enhanced plasma DHEAS, brain acetylcholine and memory mediated by steroid sulfatase inhibition. Brain Res. 1997;773(1‐2):28–32. 10.1016/s0006-8993(97)00867-6 9409701

[49] Doherty MJ , Glass IA , Bennett CL , Cotter PD , Watson NF , Mitchell AL , et al. An Xp; Yq translocation causing a novel contiguous gene syndrome in brothers with generalized epilepsy, ichthyosis, and attention deficits. Epilepsia. 2003;44(12):1529–35. 10.1111/j.0013-9580.2003.61702.x 14636323

[50] Tobias ES , Bryce G , Farmer G , et al. Absence of learning difficulties in a hyperactive boy with a terminal Xp deletion encompassing the MRX49 locus. J Med Genet. 2001;38(7):466–70. 10.1136/jmg.38.7.466 11474655 PMC1757174

[51] Thapar A , Cooper M . Attention deficit hyperactivity disorder. Lancet. 2016;387(10024):1240–50. 10.1016/S0140-6736(15)00238-X 26386541

[52] Chatterjee S , Humby T , Davies W . Behavioural and psychiatric phenotypes in men and boys with X‐linked ichthyosis: evidence from a worldwide online survey. PLoS One. 2016;11(10):e0164417. 10.1371/journal.pone.0164417 27711218 PMC5053497

[53] Sedgwick JA , Merwood A , Asherson P . The positive aspects of attention deficit hyperactivity disorder: a qualitative investigation of successful adults with ADHD. Atten Defic Hyperact Disord. 2019;11(3):241–53. 10.1007/s12402-018-0277-6 30374709

[54] Myers KA , Simard‐Tremblay E , Saint‐Martin C . X‐linked familial focal epilepsy associated with Xp22.31 deletion. Pediatr Neurol. 2020;108:113–16. 10.1016/j.pediatrneurol.2020.02.008 32299744

[55] Ozawa H , Osawa M , Nagai T , Sakura N . Steroid sulfatase deficiency with bilateral periventricular nodular heterotopia. Pediatr Neurol. 2006;34(3):239–41. 10.1016/j.pediatrneurol.2005.08.015 16504797

[56] Stergiakouli E , Langley K , Williams H , Walters J , Williams NM , Suren S , et al. Steroid sulfatase is a potential modifier of cognition in attention deficit hyperactivity disorder. Gene Brain Behav. 2011;10(3):334–44. 10.1111/j.1601-183X.2010.00672.x PMC3664024 21255266

[57] Humby T , Fisher A , Allen C , Reynolds M , Hartman A , Giegling I , et al. A genetic variant within STS previously associated with inattention in boys with attention deficit hyperactivity disorder is associated with enhanced cognition in healthy adult males. Brain Behav. 2017;7(3):e00646. 10.1002/brb3.646 28293481 PMC5346528

[58] Davies W , Humby T , Isles AR , Burgoyne PS , Wilkinson LS . X‐monosomy effects on visuospatial attention in mice: a candidate gene and implications for Turner syndrome and attention deficit hyperactivity disorder. Biol Psychiatr. 2007;61(12):1351–60. 10.1016/j.biopsych.2006.08.011 17161381

[59] Trent S , Cassano T , Bedse G , Ojarikre OA , Humby T , Davies W . Altered serotonergic function may partially account for behavioral endophenotypes in steroid sulfatase‐deficient mice. Neuropsychopharmacology. 2012;37(5):1267–74. 10.1038/npp.2011.314 22189290 PMC3306888

[60] Strous RD , Spivak B , Yoran‐Hegesh R , Maayan R , Averbuch E , Kotler M , et al. Analysis of neurosteroid levels in attention deficit hyperactivity disorder. Int J Neuropsychopharmacol. 2001;4(3):259–64. 10.1017/S1461145701002462 11602031

[61] Maayan R , Yoran‐Hegesh R , Strous R , Nechmad A , Averbuch E , Weizman A , et al. Three‐month treatment course of methylphenidate increases plasma levels of dehydroepiandrosterone (DHEA) and dehydroepiandrosterone‐sulfate (DHEA‐S) in attention deficit hyperactivity disorder. Neuropsychobiology. 2003;48(3):111–5. 10.1159/000073626 14586159

[62] Frau R , Traccis F , Bortolato M . Neurobehavioural complications of sleep deprivation: shedding light on the emerging role of neuroactive steroids. J Neuroendocrinol. 2020;32(1):e12792. 10.1111/jne.12792 31505075 PMC6982588

[63] Xerfan EMS , Facina AS , Tomimori J , Tufik S , Andersen ML . The importance of evaluating sleep complaints in children with ichthyoses: a commentary on physical and psychological growth impairment. J Clin Sleep Med. 2021;17(6):1147–48. 10.5664/jcsm.9208 33682676 PMC8314648

[64] Vorstman JA , Staal WG , van Daalen E , Franke L , van Engeland H . Identification of novel autism candidate regions through analysis of reported cytogenetic abnormalities associated with autism. Mol Psychiatr. 2006;11(1):18–28. 10.1038/sj.mp.4001781 16205736

[65] Zeidan J , Fombonne E , Scorah J , Ibrahim A , Durkin MS , Saxena S , et al. Global prevalence of autism: a systematic review update. Autism Res. 2022;15(5):778–90. 10.1002/aur.2696 35238171 PMC9310578

[66] Baek WS , Aypar U . Neurological manifestations of X‐linked ichthyosis: case report and review of the literature. Case Rep Genet. 2017;2017:9086408–5. 10.1155/2017/9086408 28884032 PMC5572599

[67] Malik A , Amer AB , Salama M , Haddad B , Alrifai MT , Balwi MA , et al. X‐linked ichthyosis associated with psychosis and behavioral abnormalities: a case report. J Med Case Rep. 2017;11(1):267. 10.1186/s13256-017-1420-2 28934990 PMC5609014

[68] Aaberg KM , Gunnes N , Bakken IJ , Lund Soraas C , Berntsen A , Magnus P , et al. Incidence and prevalence of childhood epilepsy: a nationwide cohort study. Pediatrics. 2017;139(5). 10.1542/peds.2016-3908 28557750

[69] Tuem KB , Atey TM . Neuroactive steroids: receptor interactions and responses. Front Neurol. 2017;8:442. 10.3389/fneur.2017.00442 28894435 PMC5581316

[70] Ohyama A , Nakano H , Imanishi Y , Seto T , Tsuruta D , Fukai K . A novel missense mutation of the STS gene in two siblings with X‐linked ichthyosis, complicated by short stature, bone density reduction, epilepsy, and cryptorchidism. Clin Exp Dermatol. 2019;44(1):78–9. 10.1111/ced.13741 30221377

[71] Rees E , Creeth HDJ , Hwu HG , Chen WJ , Tsuang M , Glatt SJ , et al. Schizophrenia, autism spectrum disorders and developmental disorders share specific disruptive coding mutations. Nat Commun. 2021;12(1):5353. 10.1038/s41467-021-25532-4 34504065 PMC8429694

[72] Rees E , Kirov G . Copy number variation and neuropsychiatric illness. Curr Opin Genet Dev. 2021;68:57–63. 10.1016/j.gde.2021.02.014 33752146 PMC8219524

[73] Milunsky J , Huang XL , Wyandt HE , Milunsky A . Schizophrenia susceptibility gene locus at Xp22.3. Clin Genet. 1999;55(6):455–60. 10.1034/j.1399-0004.1999.550610.x 10450863

[74] Buoli M , Caldiroli A , Serati M , Grassi S , Altamura AC . Sex steroids and major psychoses: which role for DHEA‐S and progesterone. Neuropsychobiology. 2016;73(3):178–83. 10.1159/000444922 27100685

[75] Davies W . Does steroid sulfatase deficiency influence postpartum psychosis risk? Trends Mol Med. 2012;18(5):256–62. 10.1016/j.molmed.2012.03.001 22475435

[76] Sun Q , Ren I , Zaki T , Maciejewski K , Choate K . Ichthyosis affects mental health in adults and children: a cross‐sectional study. J Am Acad Dermatol. 2020;83(3):951–54. 10.1016/j.jaad.2020.01.052 32006604

[77] Wren GH , Humby T , Thompson AR , Davies W . Mood symptoms, neurodevelopmental traits, and their contributory factors in X‐linked ichthyosis, ichthyosis vulgaris and psoriasis. Clin Exp Dermatol. 2022;47(6):1097–108. 10.1111/ced.15116 35104372 PMC9314151

[78] Lam S , Hultin S , Preston J , Campbell S . Temporal change in blood group after bone marrow transplant: a case of successful ABO‐incompatible deceased donor transplant. Case Rep Transplant. 2020;2020:7461052–4. 10.1155/2020/7461052 32774979 PMC7396079

[79] Wren G , Davies W . Sex‐linked genetic mechanisms and atrial fibrillation risk. Eur J Med Genet. 2022;65(4):104459. 10.1016/j.ejmg.2022.104459 35189376

[80] Martinez‐Raga J , Knecht C , Szerman N , Martinez MI . Risk of serious cardiovascular problems with medications for attention‐deficit hyperactivity disorder. CNS Drugs. 2013;27(1):15–30. 10.1007/s40263-012-0019-9 23160939

[81] Maki Y , Takeichi T , Kono M , Tanaka Y , Akiyama M . Case of mild X‐linked ichthyosis complicated with paroxysmal supraventricular tachycardia and anemia. J Dermatol. 2018;45(10):e275–e76. 10.1111/1346-8138.14307 29569268

[82] Palmieri C , Szydlo R , Miller M , Barker L , Patel NH , Sasano H , et al. IPET study: an FLT‐PET window study to assess the activity of the steroid sulfatase inhibitor irosustat in early breast cancer. Breast Cancer Res Treat. 2017;166(2):527–39. 10.1007/s10549-017-4427-x 28795252 PMC5668341

[83] Boe C , Blazar P , Iannuzzi N . Dupuytren contractures: an update of recent literature. J Hand Surg Am. 2021;46(10):896–906. 10.1016/j.jhsa.2021.07.005 34452797

[84] Brcic L , Wren GH , Underwood JFG , Kirov G , Davies W . Comorbid medical issues in X‐linked ichthyosis. JID Innov. 2022;2(3):100109. 10.1016/j.xjidi.2022.100109 35330591 PMC8938907

[85] Mayo Clinic . Acitretin (oral route). Available from: https://www.mayoclinic.org/drugs‐supplements/acitretin‐oral‐route/side‐effects/drg‐20061491. Accessed 8 September 2022.

[86] Sanchez LD , Pontini L , Marinozzi M , Sanchez‐Aranguren LC , Reis A , Dias IH . Cholesterol and oxysterol sulfates: pathophysiological roles and analytical challenges. Br J Pharmacol. 2021;178(16):3327–41. 10.1111/bph.15227 32762060

[87] Hoppe T , Winge MC , Bradley M , Nordenskjold M , Vahlquist A , Berne B , et al. X‐linked recessive ichthyosis: an impaired barrier function evokes limited gene responses before and after moisturizing treatments. Br J Dermatol. 2012;167(3):514–22. 10.1111/j.1365-2133.2012.10979.x 22486194

[88] Smyth N , Vatansever HS , Murray P , Meyer M , Frie C , Paulsson M , et al. Absence of basement membranes after targeting the LAMC1 gene results in embryonic lethality due to failure of endoderm differentiation. J Cell Biol. 1999;144(1):151–60. 10.1083/jcb.144.1.151 9885251 PMC2148127

[89] Hu L , Fan X , Lin J , Liang F , Tu J , Guo Z . Integrated bioinformatics analysis reveals significant genes associated with cryptorchidism (CO). Res Square. 10.21203/rs.3.rs-1690590/v1

[90] Neumann JC , Dovey JS , Chandler GL , Carbajal L , Amatruda JF . Identification of a heritable model of testicular germ cell tumor in the zebrafish. Zebrafish. 2009;6(4):319–27. 10.1089/zeb.2009.0613 20047465 PMC2811880

[91] Saikia P , Medeiros CS , Thangavadivel S , Wilson SE . Basement membranes in the cornea and other organs that commonly develop fibrosis. Cell Tissue Res. 2018;374(3):439–53. 10.1007/s00441-018-2934-7 30284084 PMC6258348

[92] Afshari NA , Igo RP, Jr. , Morris NJ , Stambolian D , Sharma S , Pulagam VL , et al. Genome‐wide association study identifies three novel loci in Fuchs endothelial corneal dystrophy. Nat Commun. 2017;8(1):14898. 10.1038/ncomms14898 28358029 PMC5379100

[93] Halfter W , Dong S , Yip YP , Willem M , Mayer U . A critical function of the pial basement membrane in cortical histogenesis. J Neurosci. 2002;22(14):6029–40. 10.1523/JNEUROSCI.22-14-06029.2002 12122064 PMC6757907

[94] De Angelis C , Byrne AB , Morrow R , Feng J , Ha T , Wang P , et al. Compound heterozygous variants in LAMC3 in association with posterior periventricular nodular heterotopia. BMC Med Genom. 2021;14(1):64. 10.1186/s12920-021-00911-4 PMC7916305 33639934

[95] van der Meer D , Kaufmann T , Shadrin AA , Makowski C , Frei O , Roelfs D , et al. The genetic architecture of human cortical folding. Sci Adv. 2021;7(51):eabj9446. 10.1126/sciadv.abj9446 34910505 PMC8673767

[96] Shadrin AA , Kaufmann T , van der Meer D , Palmer CE , Makowski C , Loughnan R , et al. Vertex‐wise multivariate genome‐wide association study identifies 780 unique genetic loci associated with cortical morphology. Neuroimage. 2021;244:118603. 10.1016/j.neuroimage.2021.118603 34560273 PMC8785963

[97] Hoogman M , van Rooij D , Klein M , Boedhoe P , Ilioska I , Li T , et al. Consortium neuroscience of attention deficit/hyperactivity disorder and autism spectrum disorder: the ENIGMA adventure. Hum Brain Mapp. 2022;43(1):37–55. 10.1002/hbm.25029 32420680 PMC8675410

[98] Gong Q , Lui S , Sweeney JA A selective review of cerebral abnormalities in patients with first‐episode schizophrenia before and after treatment. Am J Psychiatr. 2016;173(3):232–43. 10.1176/appi.ajp.2015.15050641 26621570

[99] Boland E , Quondamatteo F , Van Agtmael T The role of basement membranes in cardiac biology and disease. Biosci Rep. 2021;41(8). 10.1042/BSR20204185 PMC8390786 34382650

[100] Wang J , Xie L , Chen X , Lyu P , Zhang Q . Changes in laminin in acute heart failure. Int Heart J. 2022;63(3):454–58. 10.1536/ihj.21-769 35650146

[101] Liu B , Li X , Zhao C , Wang Y , Lv M , Shi X , et al. Proteomic analysis of atrial appendages revealed the pathophysiological changes of atrial fibrillation. Front Physiol. 2020;11:573433. 10.3389/fphys.2020.573433 33041871 PMC7526521

[102] Huang J , Luo R , Zheng C , Cao X , Zhu Y , He T , et al. Integrative analyses identify potential key genes and calcium‐signaling pathway in familial atrioventricular nodal reentrant tachycardia using whole‐exome sequencing. Front Cardiovasc Med. 2022;9:910826. 10.3389/fcvm.2022.910826 35924220 PMC9339905

[103] Derrick CJ , Pollitt EJG , Sanchez Sevilla Uruchurtu A , Hussein F , Grierson AJ , Noel ES . Lamb1a regulates atrial growth by limiting second heart field addition during zebrafish heart development. Development. 2021;148(20). 10.1242/dev.199691 34568948

[104] Malan D , Reppel M , Dobrowolski R , Roell W , Smyth N , Hescheler J , et al. Lack of laminin gamma1 in embryonic stem cell‐derived cardiomyocytes causes inhomogeneous electrical spreading despite intact differentiation and function. Stem Cell. 2009;27(1):88–99. 10.1634/stemcells.2008-0335 18927478

[105] Viil J , Maasalu K , Maemets‐Allas K , Tamming L , Lohmussaar K , Tooming M , et al. Laminin‐rich blood vessels display activated growth factor signaling and act as the proliferation centers in Dupuytren's contracture. Arthritis Res Ther. 2015;17(1):144. 10.1186/s13075-015-0661-y 26018562 PMC4475288

[106] Alfonso‐Rodriguez CA , Garzon I , Garrido‐Gomez J , Oliveira ACX , Martin‐Piedra MA , Scionti G , et al. Identification of histological patterns in clinically affected and unaffected palm regions in dupuytren's disease. PLoS One. 2014;9(11):e112457. 10.1371/journal.pone.0112457 25379672 PMC4224499

[107] Magro G , Fraggetta F , Travali S , Lanzafame S . Immunohistochemical expression and distribution of alpha2beta1, alpha6beta1, alpha5beta1 integrins and their extracellular ligands, type IV collagen, laminin and fibronectin in palmar fibromatosis. Gen Diagn Pathol. 1997;143(4):203–8. 9489951

[108] Chen ZL , Yao Y , Norris EH , Kruyer A , Jno‐Charles O , Akhmerov A , et al. Ablation of astrocytic laminin impairs vascular smooth muscle cell function and leads to hemorrhagic stroke. J Cell Biol. 2013;202(2):381–95. 10.1083/jcb.201212032 23857767 PMC3718965

[109] Humby T , Cross ES , Messer L , Guerrero S , Davies W . A pharmacological mouse model suggests a novel risk pathway for postpartum psychosis. Psychoneuroendocrinology. 2016;74:363–70. 10.1016/j.psyneuen.2016.09.019 27728876 PMC5094271

[110] Negishi Y , Nomizu M . Laminin‐derived peptides: applications in drug delivery systems for targeting. Pharmacol Ther. 2019;202:91–7. 10.1016/j.pharmthera.2019.05.017 31158392

[111] Jun JI , Lau LF . Taking aim at the extracellular matrix: CCN proteins as emerging therapeutic targets. Nat Rev Drug Discov. 2011;10(12):945–63. 10.1038/nrd3599 22129992 PMC3663145

[112] Jarvelainen H , Sainio A , Koulu M , Wight TN , Penttinen R . Extracellular matrix molecules: potential targets in pharmacotherapy. Pharmacol Rev. 2009;61(2):198–223. 10.1124/pr.109.001289 19549927 PMC2830117

